# The determination of the boundaries and prediction the radicality of glioblastoma resection using MRI and CT perfusion

**DOI:** 10.3389/fneur.2025.1572845

**Published:** 2025-05-14

**Authors:** Rustam Talybov, Tatiana Trofimova, Vadim Mochalov, Sergey Karasev, Vladislava Gorshkova, Tatiana Kleschevnikova, Irina Karasyova, Artem Batalov, Natalia Zakharova, Elena Gaijsina, Igor Pronin

**Affiliations:** ^1^Regional Clinical Hospital, Tyumen, Russia; ^2^Department of Oncology, Tyumen State Medical University of the Ministry of Healthcare of Russia, Tyumen, Russia; ^3^Department of Radiology, First Pavlov State Medical University of St. Petersburg, St. Petersburg, Russia; ^4^Department of Neurosurgery Federal Center of Neurosurgery, Tyumen, Russia; ^5^Federal State Autonomous Institution “N.N. Burdenko National Medical Research Center of Neurosurgery” of the Ministry of Health of the Russian, Moscow, Russia; ^6^Multidisciplinary Clinical Medical Center “Medical City”, Tyumen, Russia

**Keywords:** glioblastoma, perfusion, tumor boundaries, residual tumor volume, cytoreduction volume

## Abstract

**Background:**

Preoperative identification of diffuse glioma boundaries remains an unsolved problem of modern neurooncology. The main problem is the heterogeneity of the tumor being manifested by simultaneous presence of both contrast-enhancing and non-contrasting but hyperperfused regions on imaging. Perfusion technologies are known to be a reliable tool in identifying areas with intact BBB and increased proliferative activity of vascular endothelium.

**Aim:**

The purpose of this study is to evaluate the impact of MRI and CT perfusion data in preoperative planning of surgical resection in order to achieve the maximum volume of cytoreduction and to prolong relapse-free period.

**Methods:**

The study included 74 patients with the morphologically and immunohistochemically verified diagnosis of “glioblastoma NOS.” The patients were divided into 2 groups depending on the perfusion data and the extent of tumor resection. Group 1 of patients had a surgery with the preoperative use of perfusion techniques and the resection of the contrast-enhancing and hyperperfused portion of the tumor (*n* = 42), group 2 of patients had a surgery with preoperative use of perfusion techniques and resection of only the contrast-enhancing component of the tumor (*n* = 32).

**Results:**

The results of the study show that the surgery directed to the resection of contrast-positive and hyperperfused tumor portions has an advantage when compared with surgery aimed at removing only the contrast-enhancing part of the tumor. In group 1, the median relapse-free period was 15 months, while the relapse-free survival in 6 and 12 months was 92 and 55% which exceeded the results in the second group, in which the median was 9 months, and the relapse-free survival in 6 and 12 months was 66 and 9% (*p* < 0.001).

**Conclusion:**

Our study shows that the use of perfusion techniques in preoperative planning of the resection volume has a favorable potential and high diagnostic value. Perfusion tools may be contribute to the most objective assessment of all tumor components. The prolongation of the relapse-free period was achieved by taking into account the factor as the resection of both the contrast-enhanced component and the contrast-negative component with high vascular permeability detected by perfusion techniques.

## 1 Introduction

The key task of surgical treatment of glioblastomas is to ensure minimal residual tumor volume. The extent of surgical resection is limited by the infiltrative nature of glioma and the associated difficulties in assessing its boundaries. In order to achieve the maximum possible cytoreduction, the search for the most effective method of pre- and intraoperative navigation assistance is still ongoing (1–3). Malignant gliomas including in particular glioblastomas are characterized by a unique histoanatomical structure consisting of the simultaneous presence of malignant areas with both impaired and intact blood–brain barrier. The most aggressive regions are distinguished by high values of hemodynamic biomarkers: tumor blood volume (TBV) and tumor blood flow (TBF) as well as their normalized indicators: normalized blood volume (nTBV) and normalized blood flow (nTBF). Hemodynamic parameters are calculated using perfusion tools in computed tomography (CT) and magnetic resonance imaging (MRI) (4–6). CT and MRI perfusion techniques are interchangeable which have a high sensitivity and specificity for assessing the blood flow of malignant gliomas and they are able to depict qualitative and quantitative values of tumor hemodynamic parameters: TBF, TBV, nTBF and nTBV (7–9). According to numerous studies diffuse gliomas with high nTBV values are more malignant (the normalized value of volumetric cerebral blood flow for glioblastomas should be by 5 times higher than the value of intact white matter of the contralateral hemisphere) (10–13). In addition, the areas of microvascular proliferation are located not only in the contrast-enhancing part of the tumor, but also along the periphery, in the area of a combination of perifocal edema and infiltration, which are not distinguishable according to the results of post-contrast T1 series (3, 14). In our work we analyzed the effectiveness of CT and MRI perfusion techniques in preoperative determination of more malignant regions of the tumor and their boundaries and the effect of preoperative resection planning on the relapse-free patients` lifespan.

## 2 Materials and methods

### 2.1 Patients and sampling

Seventy-four patients at the age of 20 to 76 with a histologically and immunohistochemically verified diagnosis of glioblastoma were included in the study. The tumor status of isocitrate dehydrogenase (IDH) gene expression was not taken into account with the further assignment of the “not otherwise specified” (NOS) type. All tumors had a unilateral localization. None of the patients had undergone surgery before being included in the study. All patients underwent surgery at the Federal Center for Neurosurgery from 2018 to 2021. All patients had a postoperative chemotherapy and radiation therapy according to the Stupp-protocol based on data obtained during the immunohistochemical verification. The protocol included 2 steps: a six-week course with the combination of radiation therapy for 5 days with 2 Gray per fraction (session) and chemotherapy with Temozolomide continuously at 75 mg/m2, and a six-week course of adjuvant therapy with Temozolomide 150–200 mg/m2 for 5 days per month after a four-week break.

### 2.2 Multiparametric CT and MRI features

All patients included in the study underwent an MRI examination on the General Electric Discovery 3.0 T scanner using the following pulse sequences: T1WI, T2WI, T2-Flair, DWI (with ADC maps) with a slice thickness of 5 mm, DSC-T2* perfusion with parameters: T2* GRE with the coverage of the entire brain volume, with a slice thickness of 6 mm; FOV = 240×240 mm; 96×96 matrix, TR – 1,250 ms; TE – 45.0 ms; NEX = 1; pixel bandwidth – 250.0 Hz/pixel, phase per location 34. In cases where limitations of the DSC-T2* perfusion technique related to motion artifacts and magnetic susceptibility artifacts were identified, patients additionally underwent dynamic CT perfusion.

CT perfusion was performed using a 640-slice computed tomography scanner Canon Aquilion One model with the Bayesian algorithm. The perfusion examination parameters are the following: slice thickness 5 mm, 80 kV, 60 mA, tube rotation time 0.5 s with coverage of the entire brain volume.

The contrast agent for MRI examination was Gadovist 1.0 (Gadovist, Bayer) at the rate of 0.1 mL per 1 kg of the body weight. The contrast agent for CT examination was Ultravist–370 (Ultravist, Bayer) with an injection volume of 50 mL of contrast agent and 30 mL of 0.9% saline.

### 2.3 Image processing technique

**Figure 1:**
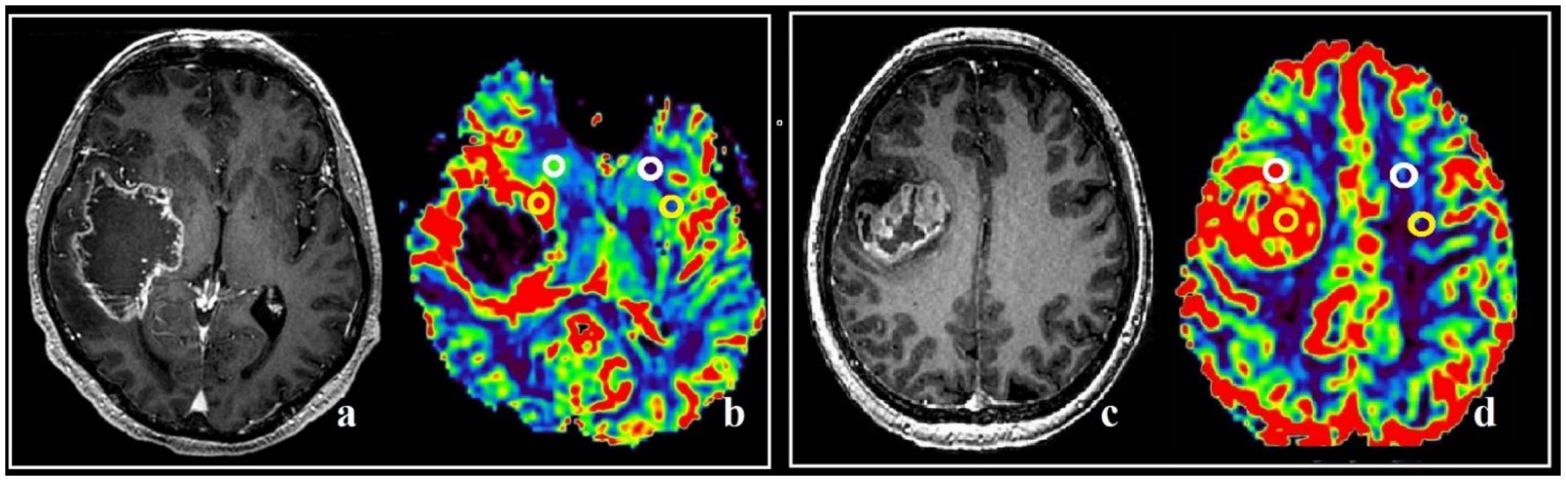
Post-contrast T1 WI and MRI, and CT-perfusion of different patients **(a–d)**. ROI segmentation with altered (yellow circles) and intact (white circles) BBB during comparison with post-contrast T1 series and DSC-T2* perfusion CBV map **(a,b)**, and post-contrast T1 series with CT-perfusion CBV map **(c,d)**. The parameters analysis was conducted both in affected and in specular contralateral hemisphere white matter.

The maximum tumor blood flow was measured using colored perfusion maps to assess the areas of the tumor to be resected, as well as the postoperative assessment of the resection degree. In hyperperfused zones a region of interest (ROI) with the area of 20–30 m2 was placed. Next, the ROI with the highest values of mean tumor blood flow and volume (TBF, TBV) in the contrast-enhancing and the contrast-negative portions of the tumor was selected for analysis. To obtain the normalized values (nTBF and nTBV), blood flow and volume in the contralateral white matter were measured in the area located contra-laterally relative to the tumor using a ROI of the same area (Figure 1). In all cases there were no regions of altered MRI signal in the contralateral hemisphere.

In all patients included in this investigation the hyperperfused portions were located both inside and outside contrast—enhancing parts. Both marked portions were inside the zone of the high signal in Т2flair.

The size of the tumor was estimated according to preoperative MRI data using contrast enhancement and DSC perfusion. Taking into account the sizes the following subgroups were marked: tumor nodes ≤50 mm at the maximum linear size in the axial plane were included in the first subgroup, the tumor nodes >50 mm were in the second subgroup.

The results of MR perfusion were analyzed in the GE ReadyView 11.3 software system, CT perfusion was analyzed in the Vitrea 4.6 (Canon Medical Systems) software system using the CT Brain Perfusion software package with the assessment of biomarkers similar to DSC-T2* perfusion.

Prior to surgery, DSC-T2* was performed in all observed patients (*n* = 74). In 23 patients the study protocol was supplemented with CT perfusion due to artifacts from blood breakdown products. In the first 48 h after surgery all patients underwent an MRI scan with DSC-T2* perfusion. In the future dynamic monitoring was performed with the accomplishment of the DSC-T2* perfusion technique in 3-month interval.

The algorithm of the preoperative planning of surgical resection using perfusion techniques was assumed to have 3 stages.

**The first stage**. MRI is performed with intravenous (IV) contrast and DSC-perfusion and/or CT-perfusion and acquired data are analyzed.

**The second stage**. Preoperative post-contrast T1 series are compared with perfusion maps, the identification of tumor boundaries and cytoreduction volume planning are determined.

**The third stage**. Performing of postoperative MRI with IV contrast and DSC-perfusion with further evaluation of residual tumor volume.

### 2.4 Stages of surgery management

Before surgery the areas of interest were coordinated by the radiologist and the neurosurgeon with the absolute exclusion of functionally significant areas. Then MRI and CT data were integrated into the neuronavigation system. In all 74 patients tumor removal was performed using standard surgical instruments with a microscope and a neural navigation system. Before the tumor resection through a small trepanation window, tissue materials were obtained from the regions of interest corresponding to the areas with impaired and intact BBB. Neuronavigation control was performed using the Stryker frameless navigation system (Stryker NAV3i, Germany).

### 2.5 Morphological and immunohistochemical studies

The verification of malignant gliomas was performed using histological and immunohistochemical methods. The immunohistochemical analysis included routine staining such as Carazzi hematoxylin and eosin alcohol solution. The spectrum of used immunohistochemical (IHC) markers are the following: CD3, CD20, CD34, Ki-67, VEGF. Additionally, all materials from the region of interest were analyzed.

The cell counting was accomplished using the Aperio ImageScope—Pathology Slide Viewing Software program using the nuclear, cytoplasmic and membrane staining analysis module—Aperio Image Analysis Workstation.

### 2.6 Statistical analysis

The statistical analysis was performed using Excel spreadsheets as well as the STATISTICA software package (version 12.0 for Windows). The comparison of independent samples by qualitative characteristics was carried out during the analysis of conjugacy tables using the Pearson’s χ2 criterion. The comparison of two groups according to quantitative characteristics with normal and abnormal distributions was performed using the Kruskal–Wallis criterion adjusted for matching values. Descriptive statistics was calculated for numerical variables (proliferative activity index values, perfusion indices TBF, TBV, nTBF, nTBV) and the nonparametric Kruskal-Wallis test was used for the intergroup comparison of proliferation indices in groups. The Kaplan-Meyer multiplicative nonparametric method was used to analyze survival rates. Cox regression was used to determine the independent factors influencing on survival rates. The correlations between variables were calculated using the Spearman coefficient. The significance level was *p < 0.05, **p < 0.01 and ***p < 0.001 for all tests.

### 2.7 The evaluation of the neurological outcomes

Karnofsky scale was used (it was carried out in the pre- and postoperative periods). The appearance / increase of focal neurological symptoms were registered over the period of patients staying at the hospital. All patients were discharged from the hospital in the satisfactory state (the state according Karnofsky scale was 70–80 points).

## 3 Results

The generalized characteristics of the patients are presented in Table 1.

**Table 1:** Patient characteristics.

Characteristics	Amount
Age group	
31–40	8
41–50	18
51–60	25
>60	23
Median (years)	57.4
Sex	
Male	46
Female	28
Ki-67%	
Low (<15%)	38
High (>15%)	36
Maximal linear tumor size on post-contrast T1 WI (cm)	
<5	29
>5	45
Tumor localization	
Confined by one lobe	50
More than one lobe engaged	24
Tumor localization	
Eloquent zone	16
Non-eloquent zone	58
Degree of resection	
Total	42
Subtotal	32

**Figure 2:**
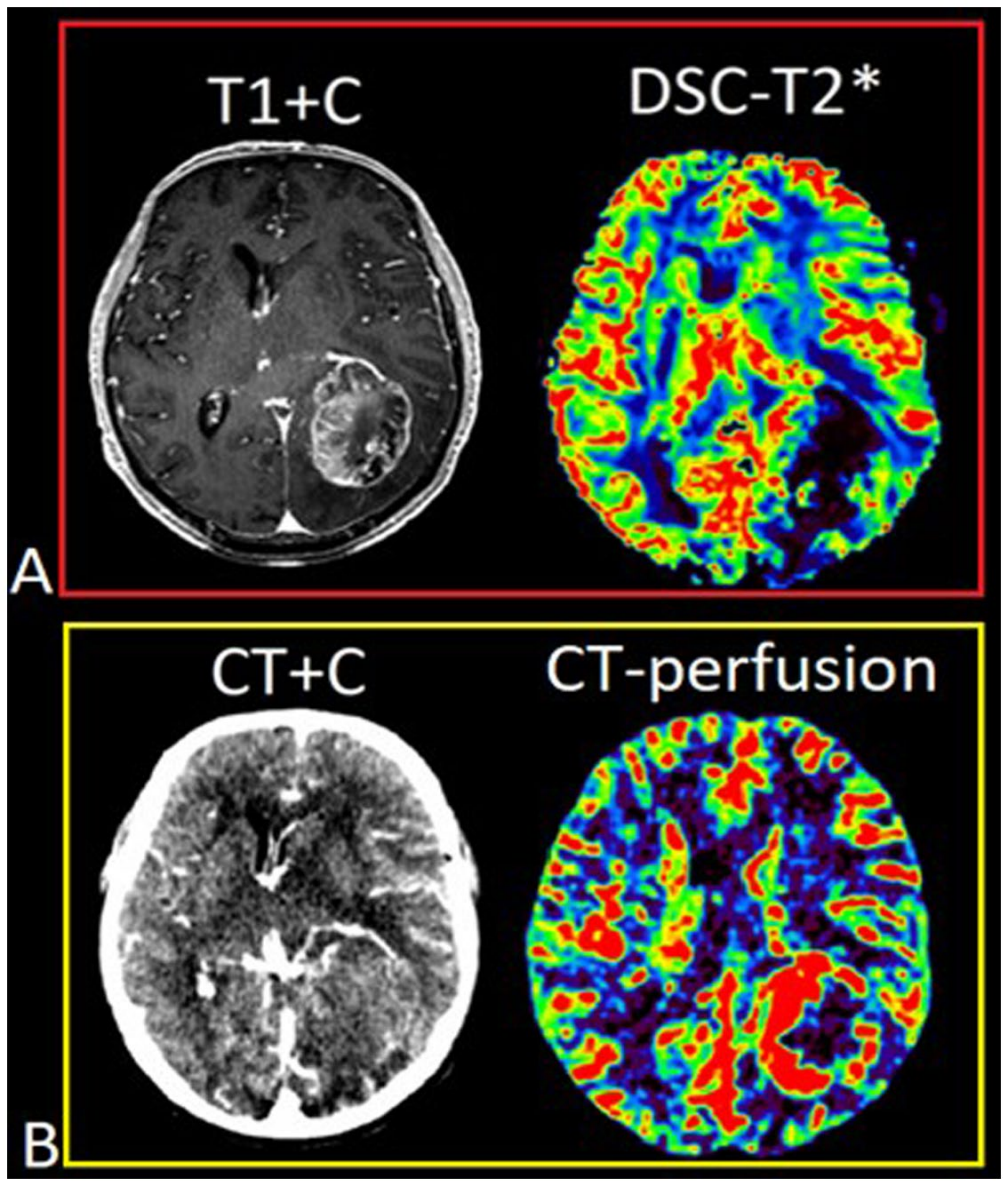
Patient T., 42 y.o., left occipital lobe glioblastoma. **(A)** Post-contrast T1 WI and DSC-T2* perfusion. DSC-T2* perfusion demonstrates marked magnetic susceptibility artifacts with local signal loss due to blood degradation products. **(В)** CT with contrast (arterial phase) and CT-perfusion. CT-perfusion does not contain any artifacts and fully displays the tumor boundaries.

DSC-T2* perfusion was performed in all 74 patients and additional CT perfusion was carried out in 23 of 74 patients. The limitations associated with artifacts from blood degradation products are shown in Figure 2.

**Figure 3:**
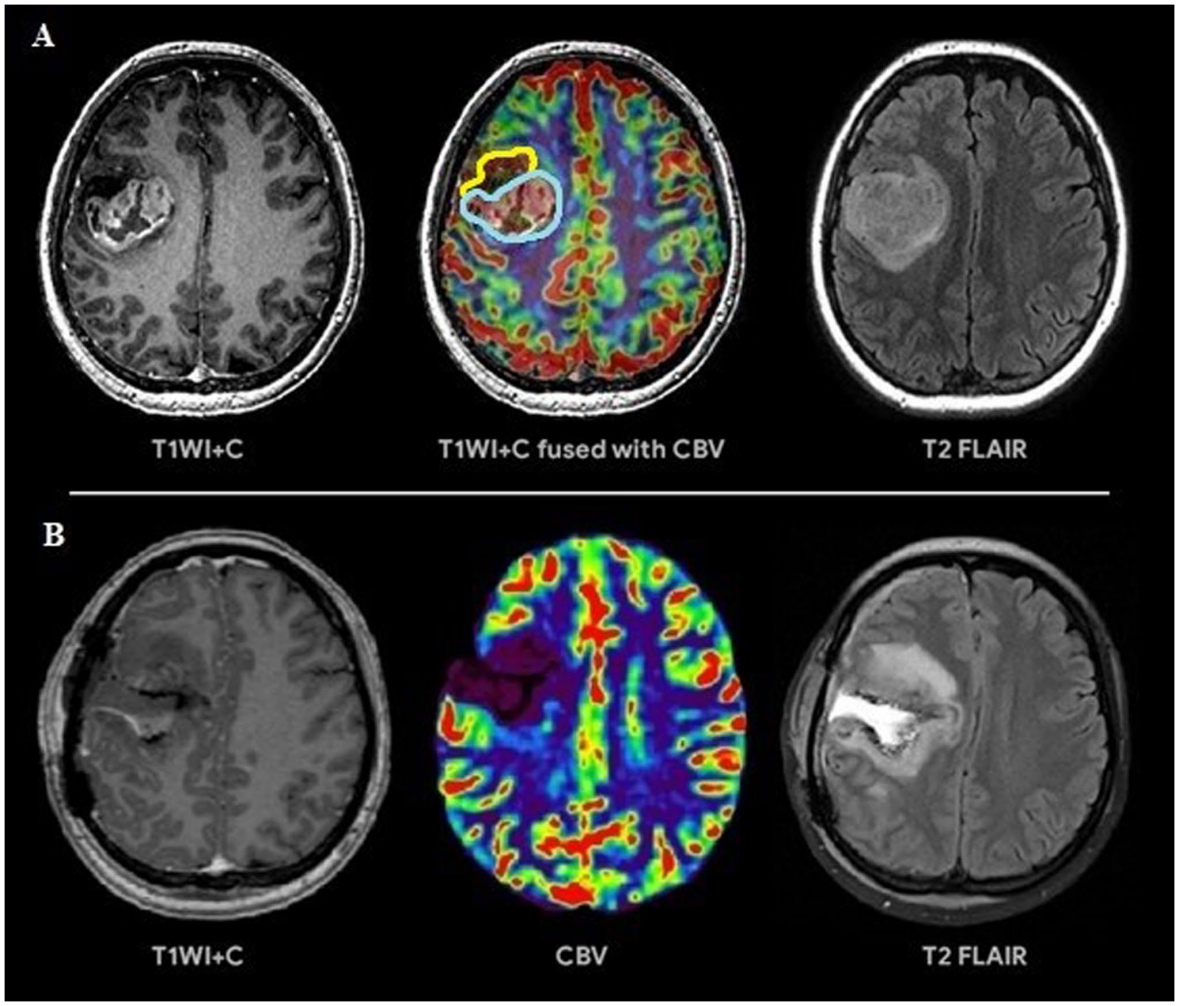
**(A)** Preoperative tumor resection planning in 42-years old patient with glioblastoma with regard to comparison of post-contrast T1 series, CBV parameter obtained using DSC-T2* perfusion and T2-Flair. The tumor has 2 portions: contrast-accumulating (blue line) and contrast-negative with high CBV volume (yellow line), which correspond to each other. **(B)** Postoperative MRI control 24 h after operation, post-contrast T1 series, CBV indicator obtained during DSC-T2* perfusion and T2-Flair. MRI data reveal resection of the contrast-accumulating component and the component with high CBV values and non-accumulating contrast agent; a zone of perifocal edema-infiltration is visualized in T2-Flair.

**Figure 4:**
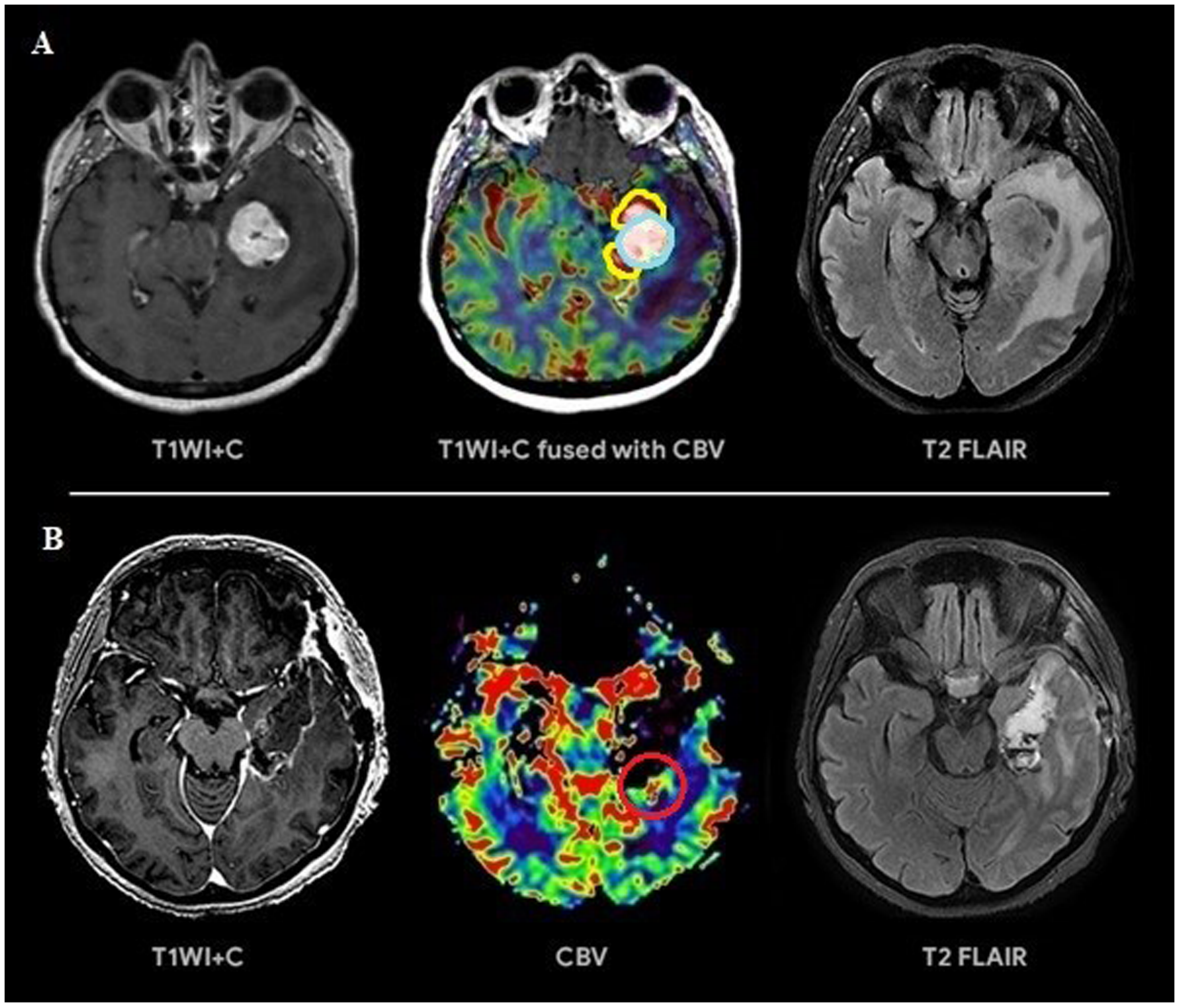
**(A)** Preoperative tumor resection planning in 52-year-old patient with left temporal lobe glioblastoma with regard to comparison of postcontrast T1 series, CBV parameter obtained using DSC-T2* perfusion and T2-Flair. The tumor compounds of 2 portions: contrast-accumulating (blue line) and contrast-negative with high CBV level (yellow line). **(B)** Postoperative MRI control 24 h after operation, post-contrast T1 series, CBV indicator obtained using DSC-T2* perfusion and T2-Flair. MRI data indicating resection of the contrast-enhancing tumor part and with residual hyperperfused fragment at the lower pole of resection zone (red circle).

Based on the conducted research the following results were obtained. Group 1 including 42 patients (56.7%; Figure 3) had a surgical resection of the contrast-enhancing and hyperperfused portions of the tumor (total resection), Group 2 including 32 people (43.3%; Figure 4) had a surgical resection of only the contrast-enhancing part of the tumor (subtotal resection).

On comparing with the classes of RANO the resection in both groups is worth estimating as class 2 (due to the contrast—enhancing component was removed except the whole Flair-component) (15). Thus, our type of resection in group 1 is the extended variant of class 2 according to RANO at the expense of hyperperfused component resection beyond the parts of accumulating contrast.

The obtained preoperative parameters of normalized tumor nTBV, nTBF in observed groups using the MRI perfusion and CT perfusion techniques are presented in Table 2 and had comparable values.

**Table 2:** Normalized perfusion parameters in glioblastomas depending on the observation group using MRI and CT perfusion.

Observational group	MRI DSC-T2* perfusion		CT-perfusion	
	nTBV	nTBF	nTBV	nTBF
Group 1	5.98 ± 1.55	8.5 ± 5.5	6.1 ± 1.45	9.35 ± 4.9
Group 2	6.01 ± 1.48	8.9 ± 4.8	6.2 ± 1.68	9.48 ± 5.3

nTBV is the mean normalized value of tumor blood volume, nTBF is the mean normalized value of tumor blood flow.

**Figure 5:**
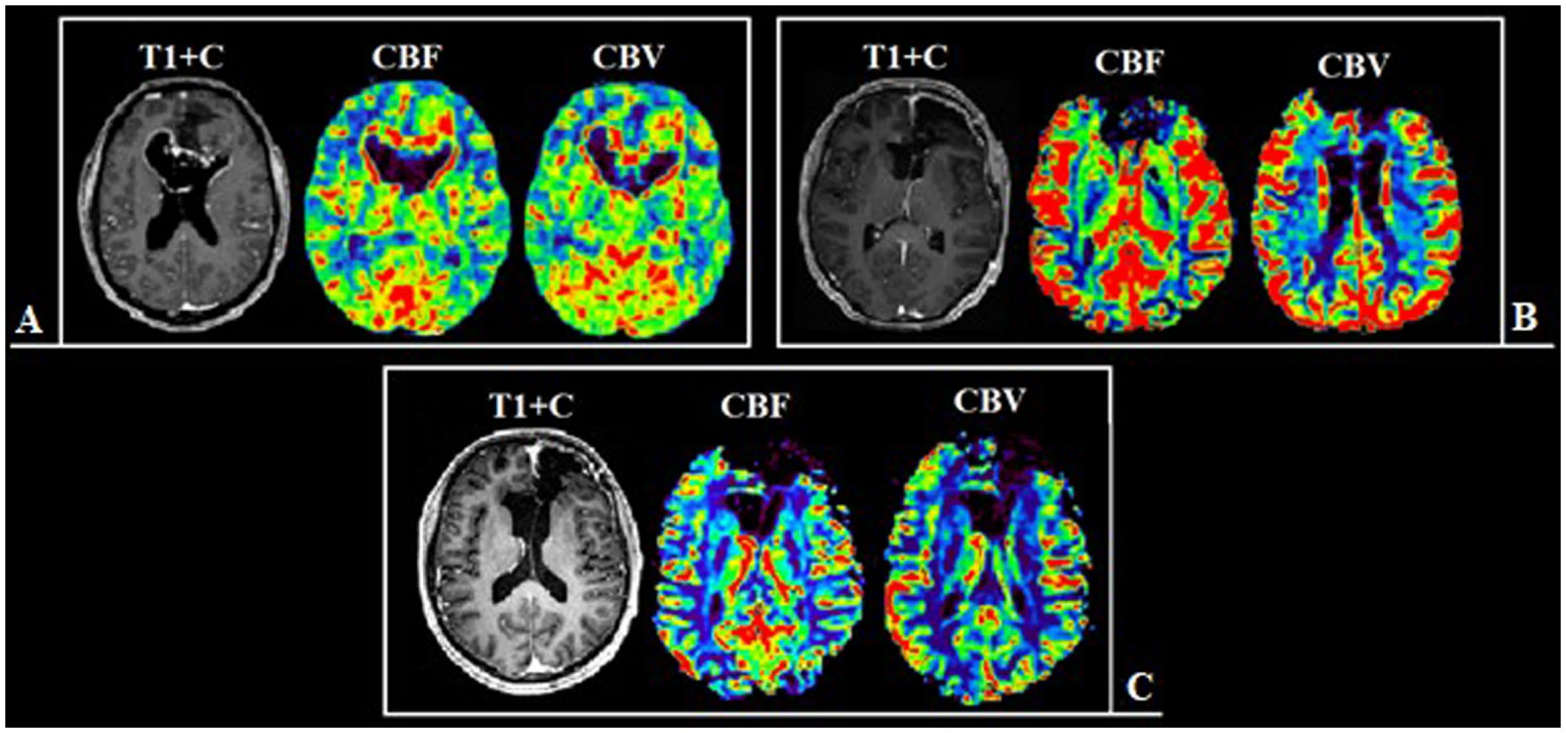
Patient N., 36-year-old, with a frontal lobes glioblastoma from group 1. **(A)** Preoperative MRI, post-contrast T1 series and CT-perfusion with CBF and CBV assessment: a tumor with characteristic “butterfly-like” contrast enhancement and central zone necrosis. The perfusion maps demonstrate high values of CBF and CBV both in the contrast-enhanced part and in the perifocal zone that does not accumulate contrast. **(B)** Postoperative MRI after 24 h, post-contrast T1 series, CBF and CBV obtainer using DSC-T2* perfusion, revealing resection of the contrast-accumulating compound and the compound with high CBV values. **(C)** Follow-up MRI 12 months after surgical treatment, post-contrast T1 series, CBF and CBV obtainer using DSC-T2* perfusion, there are no contrast-accumulating areas nor areas with high blood volume.

**Figure 6:**
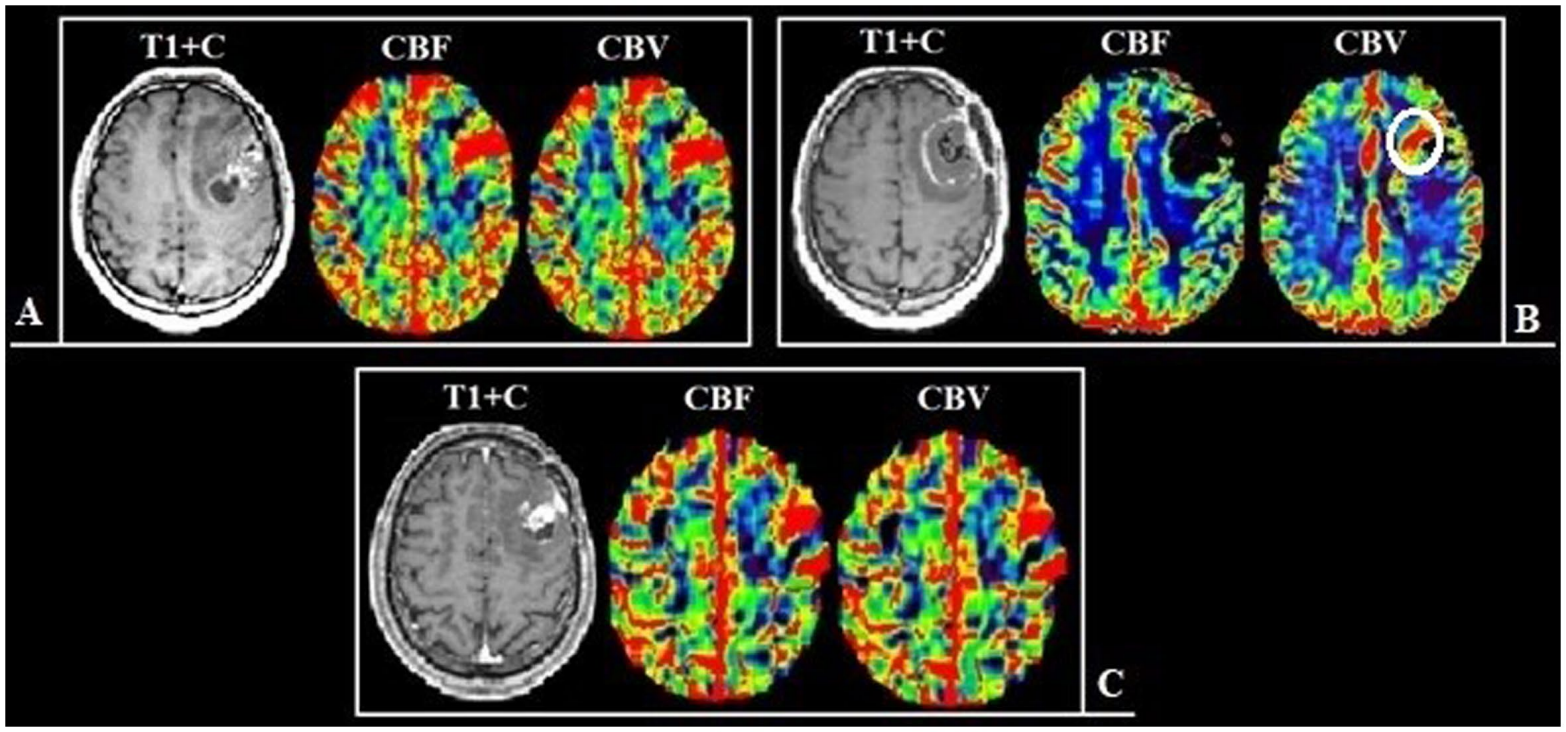
Patient V., 45-year-old, with a left frontal lobe glioblastoma from group 2. **(A)** Preoperative MRI, post-contrast T1 series and DSC-T2* perfusion: multifocal tumor with the ring-like contrast enhancement, with solid nodes and necrotic areas. The perfusion maps demonstrate high values of CBF and CBV both in the contrast-enhanced part and in the perifocal part that does not accumulate contrast. **(B)** Postoperative MRI after 24 h, post-contrast T1 series, CBF and CBV parameters obtained using DSC-T2* perfusion show the hyperperfused area localized along the medial contour of the resection (white circle). **(С)** Follow-up MRI 6 months after surgical treatment: post-contrast T1 series, CBF and CBV parameters obtained using DSC-T2* perfusion: the tumor progression in the previously identified area, which demonstrated high values of perfusion.

**Figure 7:**
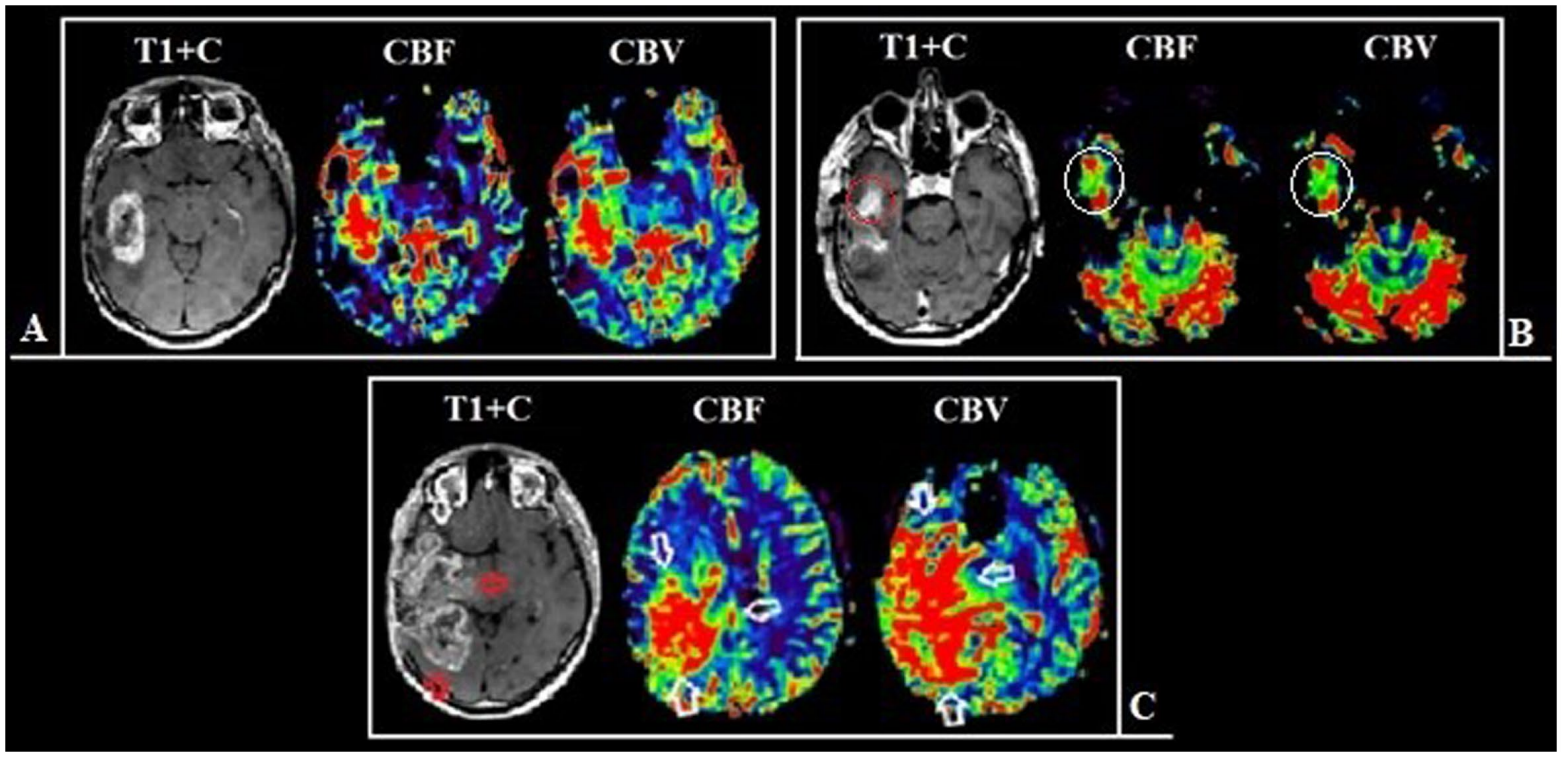
Patient Z., 56-year-old, with a right temporal lobe glioblastoma from group 2. **(A)** Preoperative MRI, post-contrast T1 series and DSC-T2* perfusion: tumor with the ring-like contrast enhancement and central necrotic area. Analyzing the perfusion maps, high CBF and CBV in contrasted tumor part and in area of edema and infiltration are noted. **(B)** Postoperative MRI after 24 h show the residual tumor parts along the resection border, which accumulate the contrast material, as well as areas demonstrating high perfusion values **(С)** Follow-up MRI 3 months after surgical treatment: the tumor show pronounced progression of both the contrast-positive (red arrow) and contrast-negative (white arrow) portions.

We identified statistically significant differences in the duration of the relapse-free period depending on the residual tumor volume in the observed groups (*p* < 0.001). At the same time, the impact was exerted not only by the residual volume of the contrast-enhancing component but also by the residual volume of the hyperperfused tumor part (Figures 5–7).

**Figure 8:**
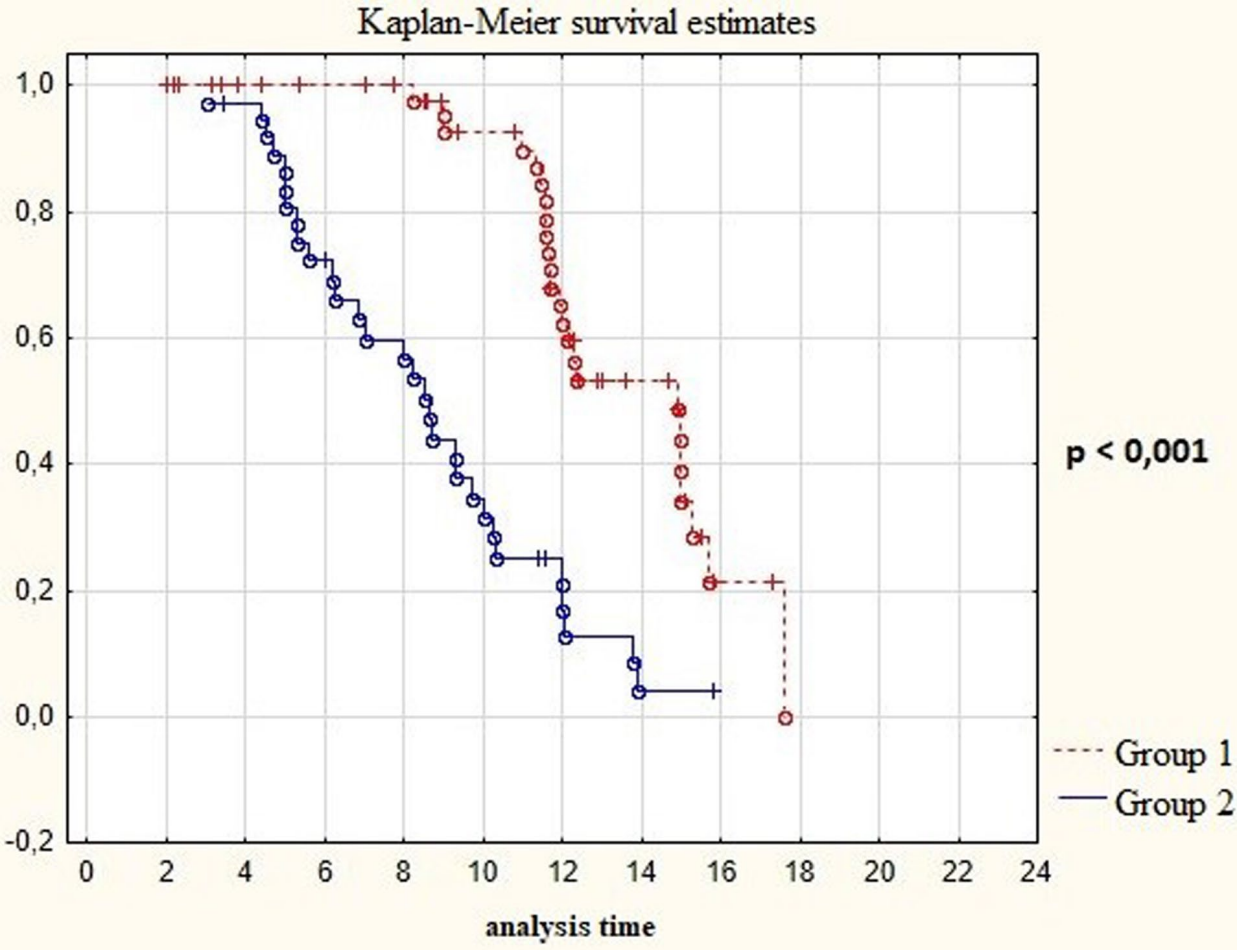
Survival time in post-operative patients with “Group 1” in comparison to “Group 2” (in months).

On analyzing Kaplan-Meyer model we obtained data indicating a prolongation of the relapse-free life expectancy of the patients during surgery with resection of the contrast-enhancing and hyperperfused tumor’s compounds. In Group 1 the median of disease–free period was up to 15 months, in Group 2 it was 9 months (Table 3; Figure 8; *p* < 0.001).

**Table 3:** Mean and median relapse-free survival rates distribution and relapse-free survival rates after 6 and 12 months in observation groups.

Observational groups	Mean of relapse-free period (months)	Median of relapse-free period (months)	Relapse-free survival rate		*р*-value
			6 months	12 months	
Group 1	13.05	15.00	92%	55%	1
Group 2	8.98	9.00	66%	9%	< 0.001

**Figure 9:**
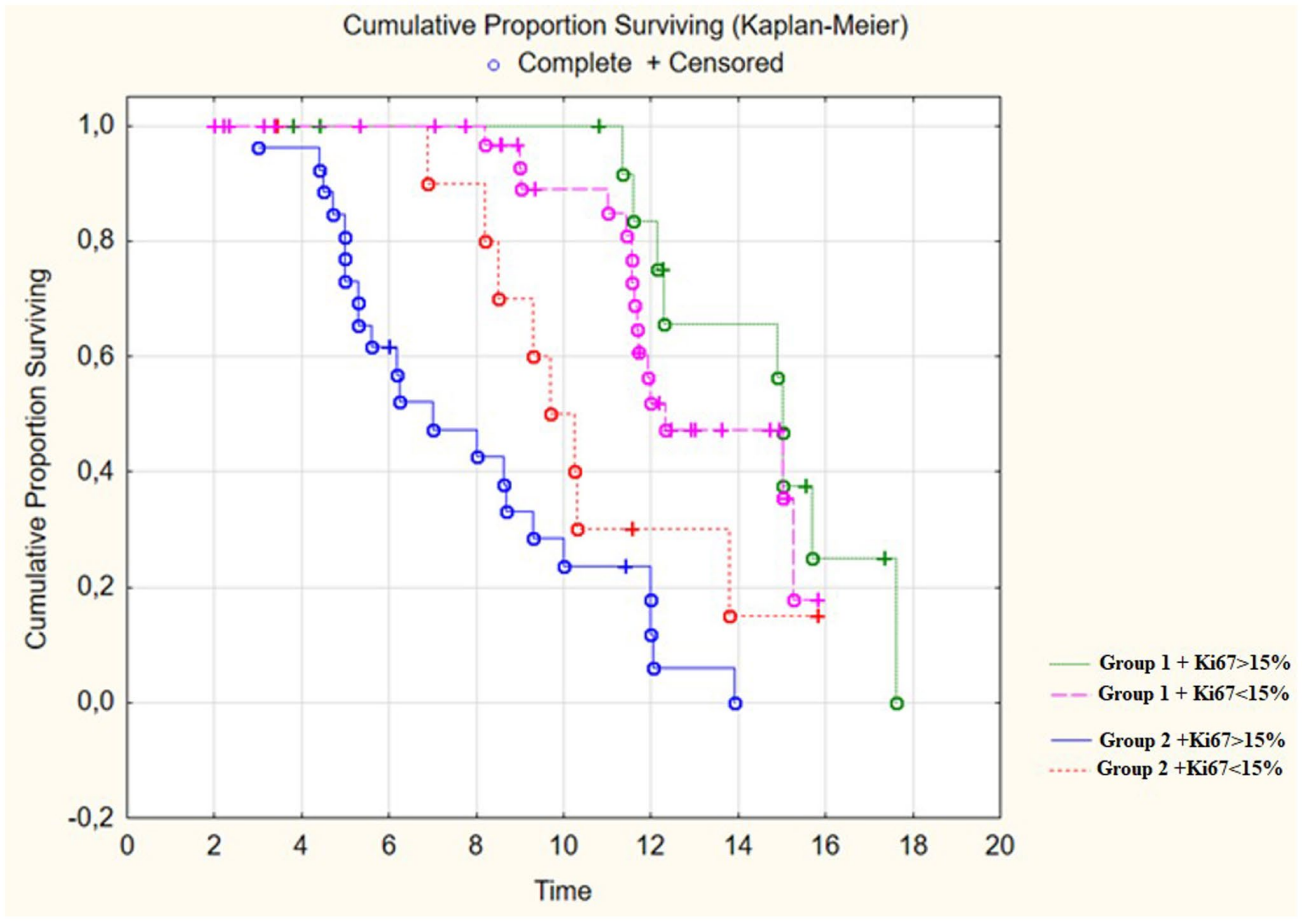
Survival Time in post-operative patients with “Group 1” in comparison to “Group 2” (in months) depending on proliferative index Ki67.

We analyzed the relationship between the duration of disease-free survival in the groups and the combination of factors which included extent of resection and the Ki-67 proliferative activity index in malignant gliomas. We found that low Ki values of 67% and the minimum residual tumor volume in the groups significantly influenced on the period of disease-free survival (*p* < 0.01; Figure 9; Table 4). In addition we analyzed the affect of the perfusion application in combination with the achievement of total resection using a multifactorial regression analysis of proportional Cox risks. The proportional risk for patients from group 2 with resection of only the contrast-accumulating portion was almost by 4 times higher than in group 1. The results are presented in Table 5.

**Table 4:** Relapse-free survival rate distribution after 6 and 12 months in observation groups depending on proliferative index Ki67.

Observed groups	Relapse-free survival rate	
	6 months	12 months
Group 1 + Ki67 < 15%	89%	48%
Group 2 + Ki67 < 15%	71%	4%
Group 1 + Ki67 > 15%	75%	18%
Group 2 + Ki67 > 15%	33%	0%

**Table 5:** Kaplan–Meier estimation, single-factor and multi-factor Cox regression analysis results.

Characteristics	Log-rank test	Amount	Univariate analysis of PFS		Multivariate analysis of PFS	
	*p*-value		HR (95% CI)	*p*-value	HR (95% CI)	*p*-value
Sex	0.123					
Male		46	1		1	
Female		28	0.77 (0.53–1.14)	0.193	0,78 (0,51-1,17)	0.216
Age (years)	0.132					
31–40					1	
41–50		8	1.82 (0.51–6.49)	0.354	2.41 (0.64–9.03)	0.192
51–60		18	1.78 (0.52–6.02)	0.356	1.99 (0.58–6.87)	0.272
>60		25	2.71 (0.82–8.97)	0.103	2.72 (0.79–9.27)	0.11
		23	2.48 (0.76–8.02)	0.132	2.69 (0.79–9.09)	0.111
Group	0.000					
Group 1		42	1	0	1	0
Group 2		32	3.54 (2.05–6.10)	0	3,68 (2.04–6.64)	0
Ki-67%	0.002					
Low		38	1	**0.008**	1	**0.041**
High		36	1.74 (1.15–2.63)		1.64 (1.02–2.63)	

*Bold values denote criteria with prognostic value.

## 4 Discussion

**Figure 10:**
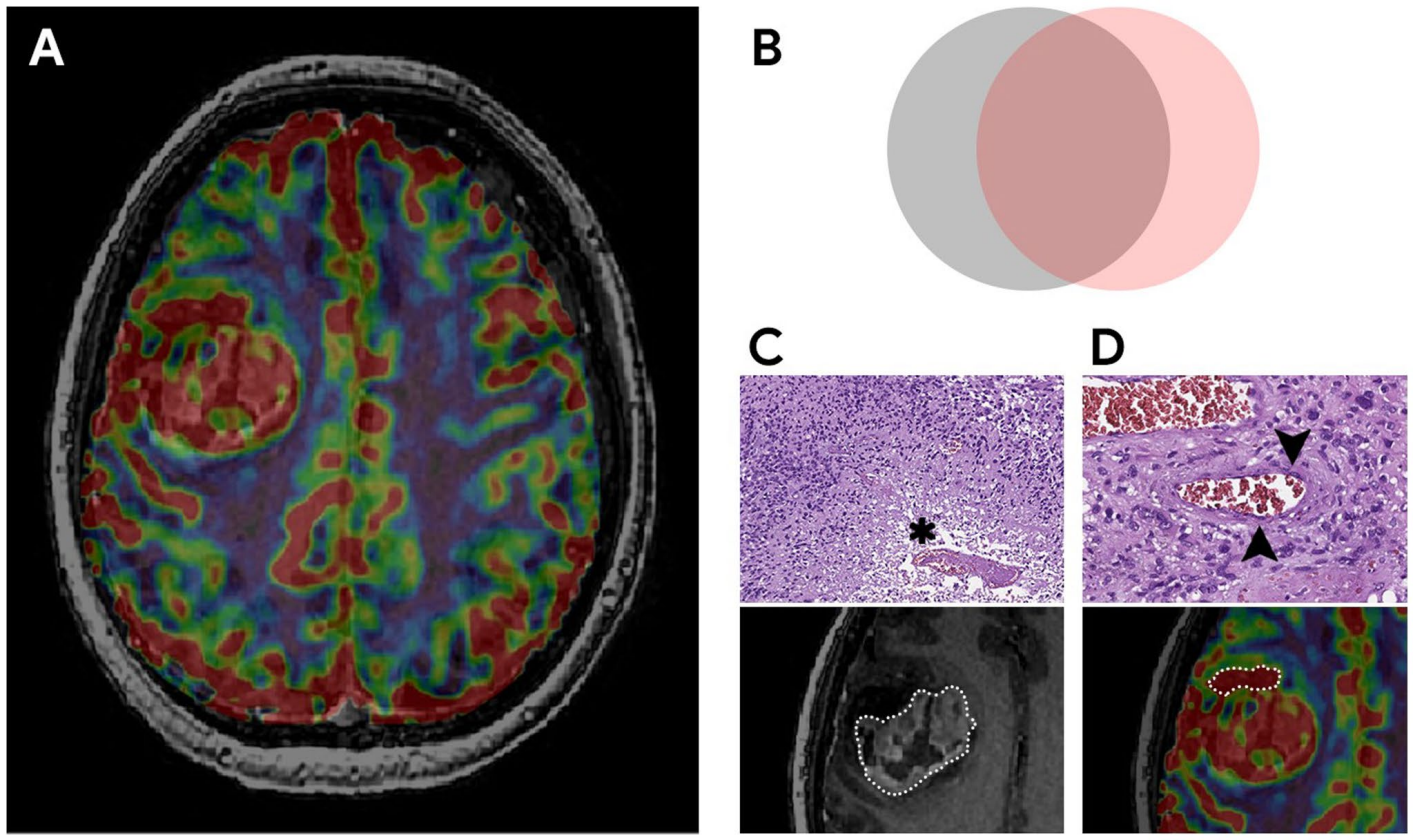
The most aggressive morphological components of glioblastoma, their relationships and pathological substrates. **(A)** MRI of the brain of a patient with right frontal lobe glioblastoma. Compared post-contrast T1 WI and CBV perfusion map, demonstrating a large contrast-enhancing component and areas with high TBV located inside and outside the high signal on the post-contrast T1 WI. **(B)** Schematic representation of the relationship between malignant components. The gray circle represents a tumor component with a disrupted BBB (the part of the tumor that accumulates contrast). The pink circle reflects the tumor component with microvascular proliferation (the part of the tumor with high TBV values). The overlap of both components represents the largest area which is coded in ivory color. The study focuses on the part with high TBV values located outside the contrast accumulation (part of the pink circle) and the investigation of the effect of its resection along with the contrast-enhancing portion on the duration of the recurrence-free period. **(C,D)** Histological examination, magnification × 10, staining with hematoxylin and eosin. Comparison of MRI data with microscopic substrates of glioblastoma radiological features. **(C)** The upper figure shows tumor cells with nuclear atypia and a large area of necrosis (asterisk), corresponding to the area of accumulation of contrast in the lower figure. The lower figure is a post-contrast T1 WI, a component of a glioblastoma with a necrosis cavity (the area highlighted by the dotted line). **(D)** The upper figure dipicts the proliferation of the endothelium of the tumor vessel (arrows), which causes high TBV values on the CBV perfusion map (lower figure). The lower figure is a CBV perfusion map, the area highlighted by the dotted line is located outside the contrasted part of the tumor.

The key factor influencing the prognosis of the disease is the extent of resection of malignant glioma (16–18, 52). It is known that overall survival is associated with total resection (total removal of the contrast-enhancing part of the tumor) which is considered to be a threshold (19). The main prognostic landmark for the effectiveness of the treatment used by neurosurgeons in practice is the residual volume of the contrast–enhancing component of diffuse glioma (20–23). However, some parts of glioblastomas are not able to accumulate the contrast agent and in rare cases, they do not accumulate it at all (24–27). The contrast-positive components of glioblastomas in the post-contrast series reflect the areas with an disrupted BBB which may or may not correspond to the most aggressive part histopathologically (28–32). Besides necrosis glioblastomas have the areas with both the increased proliferative endothelium activity in their structure and an intact BBB (33–36). The intratumoral heterogeneity of glioblastomas and the infiltrative nature of growth make it difficult to assess its boundaries (6, 19, 20, 37, 38). Surgery aimed at only resection of the contrast-enhancing part of the tumor is attended with the risk of early relapses. Implemented perfusion methods using CT and MRI have a unique ability to identify the areas with the vascular proliferation using noninvasive biomarkers of tumor hemodynamics (TBF, TBV, nTBF, nTBV). There are a number of studies examining the blood flow of a tumor in the most malignant areas (13, 39, 40). The aim of the current study is to estimate the contrast-positive and hyperperfused contrast-negative components of glioblastomas for preoperative resection volume planning. In our study data obtained are accomplished with the help of CT and MRI perfusion techniques. Perfusion parameters are taken into account both in contrast-positive components of gliomas and in components without the contrast accumulation. Besides the main stage of the tumor resection matching preoperative perfusion data including histological and immunohistochemical ones taken from the sampled tissue having the regions of interest and their interconnection is performed (Figure 10). All the samples obtained from the zone which do not accumulate the contrast agent are infiltrated by tumor cells without damage to the BBB which demonstrates the importance of perfusion CBF and CBV parameters in the contrast-negative zone and does not contradict the previously published works concerning the most malignant areas (23, 39, 40, 53).

Thus, the information given to the neurosurgeon about areas of glioma with vascular proliferation contributes to an additional and selective increase in the resection volume which, accordingly, makes it possible to predict the prolongation of patients’ life expectancy without relapse. In our study, in group with preoperative usage of perfusion mapping techniques and resection of contrast–enhancing and hyperperfused portions of the tumor, the median recurrence-free period is up to 15 months and recurrence-free survival in 6 and 12 months is up to 92 and 55%, respectively, which exceeds the recurrence-free survival periods described in the world literature which is 10.6 months with the average survival rate of 14 months (41–45).

The current study allows to assume that the practical significance of perfusion indices as biomarkers of hemodynamics of malignant gliomas remains underestimated. Perfusion data allow to plan the volume of resection individually at the preoperative stage as well as estimate more objectively the residual volume at the postoperative stage.

To determine the spread of diffuse malignant glioma MRI quantitative methods such as diffusion-weighted imaging and relaxometric are used (39).

On the basis of diffusion-weighted imaging (DWI, DTI) the gradient of the diffusion coefficient can be determined in the perifocal edema as a sign of the tumor infiltration due to solid cellularity tumor.

Diffusion kurtosis imaging (DKI) is a more sensitive method to the changes of brain tissue microstructure than a routine DWI because in this method the water diffusion in the biological tissues does not keep to Gaussian distribution (39).

The parameters of diffusion analysis are still a subject of the researches and they are not used in the daily practice due to the long term period of data receiving as well as labor and time consuming post processing (39).

Amino acid PET-CT plays a definite role in the diffuse glioma diagnostics. This method allows to grade a tumor as well its spread (46). To have a proper interpretation it is necessary to have an MRI brain preliminary data for the anatomical comparison with PET-CT structural and functional results (46). As a rule, PET-CT centers are independent structures, so a patient has to spend a lot of time for MRI, PET-CT and further neurosurgeon’s consultation to determine a tactics. This results in wasting time to begin a treatment of an aggressive tumors. There are often observations of the negative influence of radio pharmaceuticals on the brain tumor. Non-metabolic areas occur in 30% of gliomas and they do not differ from uninjured brain and it does not allow a tumor to be visualized (47, 48). There are various readings of determining a border value of the index of accumulation (49, 50). There are discrepancies in the definition of the threshold value of the accumulation index for differentiation of tumor infiltration zones and uninjured brain matter (51). Besides, the metabolic tumor volume in PET-CT with amino acids usually is not beyond the borders of zones of MRI signal on T2-FLAIR that does not allow to estimate the tumor infiltration borders as a whole (47, 48).

There is a technique called FLAIRectomy that promises high efficiency. But the ability to remove the entire T2-Flair hyperintensive zone is often not feasible (23).

We believe that a unified analysis of a complex of diffusion, perfusion parameters and PET data using labeled amino acids will allow more accurate determination of the boundaries of high-grade gliomas in areas with an intact blood–brain barrier that do not enhance. Knowledge of the extent of the tumor, in turn, will allow the development of a personalized navigation algorithm for surgical removal of the tumor with subsequent determination of an individual plan for radiation and chemotherapy, as well as the ability to predict the course of the disease.

## 5 Limitations

In the context of the 2021 WHO Classification of Tumors of the Central Nervous System in our work, we dealt with GBM or Diffuse Astrocytoma Grade 4. The histological diagnosis of malignant glioma grade 4 was made based on a paraffin section stained with hematoxylin and eosin. Given the lack of genetic research methods at the clinic’s disposal, the diagnosis was given the abbreviation “NOS.”

The study did not consider separate measurements of the volumes of the intratumoral contrast-enhancing part and the non-enhancing but hyperperfused part. The lack of this information may be a limiting factor for comparison with other studies.

The both mentioned limitations could affect on the overall survival and progression-free survival.

The technique itself has several significant limitations also. The first and the most difficult limit is the phenomenon of brain shifting after dissecting the dura mater. The shift caused by cerebrospinal fluid leakage and the changes in the intracranial pressure distort the position of the planned “coordinates” of resection. The solution of the displacement problem can be the intraoperative determination of perfusion parameters after opening the dura mater in a hybrid operating room equipped with a CT scanner on rails. The second problem is that the performance of contrast studies (DSC-perfusion and CT-perfusion) is limited after nephrectomy and the significant increase in creatinine and urea levels (glomerular filtration rate <30 mL/min).

## 6 Conclusion

The study confirms the efficiency and potential of using perfusion MRI and CT techniques in the preoperative planning of glioblastoma resection. Perfusion markers allow to determine the boundaries of the tumor objectively and evaluate its most aggressive components including both contrast-enhancing and hyperperfused areas. We have demonstrated the relationship of markers with histological and immunohistochemical data. The usage of perfusion technologies contributes to a significant increase in the volume of cytoreduction expressed in lengthening the duration of the relapse-free period. The analysis of the relationship between resection volume and disease-free survival confirm the key role of minimizing residual tumor volume in disease prognosis. In our opinion, perfusion methods have proven to be tactically significant tools in preoperative planning. We recommend integrating perfusion techniques into standard protocols for the preoperative diagnosis and surgical intervention planning for glioblastomas. The usage of perfusion in intraoperative monitoring as a navigation assistant can offset the phenomenon of displacement after opening the dura mater and support the preliminary results reliably. At the next stage of our study the significance of resection of the hypervascular component of glioblastoma will be studied using intraoperative perfusion.

## Data Availability

The original contributions presented in the study are included in the article/supplementary material. Further inquiries can be directed to the corresponding author.

## References

[1] Karschnia P Vogelbaum MA van den Bent M Cahill DP Bello L Narita Y. Evidence-based recommendations on categories for extent of resection in diffuse glioma. Eur J Cancer. (2021) 149:23–33. doi: 10.1016/j.ejca.2021.03.002, PMID: 33819718

[2] Sulangi AJ Husain A Lei H Okun J. Neuronavigation in glioma resection: current applications, challenges, and clinical outcomes. Front Surg. (2024) 11:1430567. doi: 10.3389/fsurg.2024.1430567, PMID: 39165667 PMC11334078

[3] Talybov RS Trofimova TN Mochalov VV Shvetsov IV Spasennikov VV. Intraoperative computed tomography perfusion navigation for maximal resection of high grade gliomas: a prospective non-randomized trial. Almanac Clin Med. (2023) 51:149015. doi: 10.18786/2072-0505-2023-51-012

[4] Wang K Li Y Cheng H Li S Xiang W Ming Y. Perfusion CT detects alterations in local cerebral flow of glioma related to IDH. MGMT TERT status BMC Neurol. (2021) 21:460. doi: 10.1186/s12883-021-02490-4, PMID: 34814870 PMC8611974

[5] Talybov RS Trofimova TN. Differential diagnosis of primary central nervous system lymphomas based on multiparametric MRT mapping. Diagnostic Radiol Radiotherapy. (2022) 13:36–49. doi: 10.22328/2079-5343-2022-13-2-36-49

[6] Aydin S Fatihoğlu E Koşar PN Ergün E. Perfusion and permeability MRI in glioma grading. Egypt J Radiol Nucl Med. (2020) 51:2. doi: 10.1186/s43055-019-0127-3

[7] Wu H Beylerli O Gareev I Beilerli A Ilyasova T Talybov R. Are there reliable multiparametric MRI criteria for differential diagnosis between intracranial meningiomas and solitary intracranial dural metastases? Oncol Lett. (2023) 26:350. doi: 10.3892/ol.2023.13936, PMID: 37427340 PMC10326821

[8] Herings SDA van den Elshout R de Wit R Mannil M Ravesloot C Scheenen TWJ. How to evaluate perfusion imaging in post-treatment glioma: a comparison of three different analysis methods. Neuroradiology. (2024) 66:1279–89. doi: 10.1007/s00234-024-03374-3, PMID: 38714545 PMC11246270

[9] Lee J Chen MM Liu HL Ucisik FE Wintermark M Kumar VA. MR perfusion imaging for gliomas. Magn Reson Imaging Clin N Am. (2024) 32:73–83. doi: 10.1016/j.mric.2023.07.003, PMID: 38007284

[10] Talybov RS Trofimova TN Tamrazov RI Shvetsov IV. Reliability of diffusion-weighted imaging and perfusion parameters in the differential diagnosis of malignant and considered benign intracranial tumors: a single-center study. Diagnostic Radiol Radiotherapy. (2023) 14:48–63. doi: 10.22328/2079-5343-2023-14-2-48-63

[11] Kouwenberg V van Santwijk L Meijer FJA Henssen D. Reliability of dynamic susceptibility contrast perfusion metrics in pre- and post-treatment glioma. Cancer Imaging. (2022) 22:28. doi: 10.1186/s40644-022-00466-2, PMID: 35715866 PMC9205029

[12] Batalov AI Zakharova NE Pronin IN Belyaev AY Pogosbekyan EL Goryaynov SA. 3D pCASL-perfusion in preoperative assessment of brain gliomas in large cohort of patients. Sci Rep. (2022) 12:2121. doi: 10.1038/s41598-022-05992-4, PMID: 35136119 PMC8826414

[13] Batalov AI Zakharova NE Chekhonin IV Pogosbekyan EL Sudarikova AV Goryainov SA. Arterial spin labeling perfusion in determining the IDH1 status and Ki-67 index in brain gliomas. Diagnostics. (2022) 12:1444. doi: 10.3390/diagnostics12061444, PMID: 35741254 PMC9221904

[14] Talybov RS Trofimova TN Pavlova VI Shvetsov IV Mochalov VV Martirosyan ME. Atypical case of glioblastoma localization: infratentorial localization. Radiol Prac. (2024) 1:9–19. doi: 10.52560/2713-0118-2024-1-9-19

[15] Karschnia P Young JS Dono A Häni L Sciortino T Bruno F. Prognostic validation of a new classification system for extent of resection in glioblastoma: a report of the RANO resect group. Neuro-Oncology. (2023) 25:940–54. doi: 10.1093/neuonc/noac193, PMID: 35961053 PMC10158281

[16] Hervey-Jumper SL Berger MS. Maximizing safe resection of low- and high-grade glioma. J Neuro-Oncol. (2016) 130:269–82. doi: 10.1007/s11060-016-2110-4, PMID: 27174197

[17] Kreatsoulas D Damante M Gruber M Duru O Elder JB. Supratotal surgical resection for low-grade glioma: a systematic review. Cancer. (2023) 15:2493. doi: 10.3390/cancers15092493, PMID: 37173957 PMC10177219

[18] Aziz PA Memon SF Hussain M Memon AR Abbas K Qazi SU. Supratotal resection: an emerging concept of glioblastoma Multiforme surgery-systematic review and Meta-analysis. World Neurosurg. (2023) 179:e46–55. doi: 10.1016/j.wneu.2023.07.020, PMID: 37451363

[19] Eisenbarth D Wang YA. Glioblastoma heterogeneity at single cell resolution. Oncogene. (2023) 42:2155–65. doi: 10.1038/s41388-023-02738-y, PMID: 37277603 PMC10913075

[20] Claes A Idema AJ Wesseling P. Diffuse glioma growth: a guerilla war. Acta Neuropathol. (2007) 114:443–58. doi: 10.1007/s00401-007-0293-7, PMID: 17805551 PMC2039798

[21] Kees J Gerritsen W Lianne M Broekman D De Vleeschouwer S Schucht P. Safe surgery for glioblastoma: recent advances and modern challenges. Neuro-Oncol Prac. (2022) 9:364–79. doi: 10.1093/nop/npac019, PMID: 36127890 PMC9476986

[22] Behling F Rang J Dangel E Noell S Renovanz M Mäurer I. Complete and incomplete resection for progressive glioblastoma prolongs post-progression survival. Front Oncol. (2022) 12:755430. doi: 10.3389/fonc.2022.755430, PMID: 35251956 PMC8888692

[23] Haddad AF Young JS Morshed RA Berger MS. FLAIRectomy: resecting beyond the contrast margin for glioblastoma. Brain Sci. (2022) 12:544. doi: 10.3390/brainsci12050544, PMID: 35624931 PMC9139350

[24] Lasocki A Gaillard F. Non-contrast-enhancing tumor: a new frontier in glioblastoma research. Am J Neuroradiol. (2019) 40:758–65. doi: 10.3174/ajnr.A6025, PMID: 30948373 PMC7053910

[25] Sahoo OS Mitra R Nagaiah NKH. The hidden architects of glioblastoma multiforme: glioma stem cells. MedComm Oncol. (2023) 3:1–27. doi: 10.1002/mog2.66

[26] Su CH Tsai CY Tomanek B Chen WY Cheng FY. Evaluation of blood–brain barrier-stealth nanocomposites for in situ glioblastoma theranostics applications. Nanoscale. (2016) 8:7866–70. doi: 10.1039/C6NR00280C, PMID: 27035391

[27] Duan M Cao R Yang Y Chen X Liu L Ren B. Blood-brain barrier conquest in glioblastoma nanomedicine: strategies, clinical advances, and emerging challenges. Cancer. (2024) 16:3300. doi: 10.3390/cancers16193300, PMID: 39409919 PMC11475686

[28] Ahmed MH Canney M Carpentier A Idbaih A. Overcoming the blood brain barrier in glioblastoma: status and future perspective. Rev Neurol. (2023) 179:430–6. doi: 10.1016/j.neurol.2023.03.013, PMID: 37062676

[29] Ahmed MH Canney M Carpentier A Thanou M Idbaih A. Unveiling the enigma of the blood-brain barrier in glioblastoma: current advances from preclinical and clinical studies. Curr Opin Oncol. (2023) 35:522–8. doi: 10.1097/CCO.0000000000000990, PMID: 37681417 PMC10566587

[30] Digiovanni S Lorenzati M Bianciotto OT Godel M Fontana S Akman M. Blood-brain barrier permeability increases with the differentiation of glioblastoma cells in vitro. Fluids Barriers CNS. (2024) 21:89. doi: 10.1186/s12987-024-00590-0, PMID: 39487455 PMC11529439

[31] Arvanitis CD Ferraro GB Jain RK. The blood-brain barrier and blood-tumour barrier in brain tumours and metastases. Nat Rev Cancer. (2020) 20:26–41. doi: 10.1038/s41568-019-0205-x, PMID: 31601988 PMC8246629

[32] Noorani I de la Rosa J. Breaking barriers for glioblastoma with a path to enhanced drug delivery. Nat Commun. (2023) 14:5909. doi: 10.1038/s41467-023-41694-9, PMID: 37737212 PMC10517119

[33] Agarwal A Edgar MA Desai A Gupta V Soni N Bathla G. Molecular GBM versus histopathological GBM: radiology-pathology-genetic correlation and the new WHO 2021 definition of glioblastoma. Am J Neuroradiol. (2024) 45:1006–12. doi: 10.3174/ajnr.A8225, PMID: 38438167 PMC11383408

[34] Lan Z Li X Zhang X. Glioblastoma: an update in pathology, molecular mechanisms and biomarkers. Int J Mol Sci. (2024) 25:3040. doi: 10.3390/ijms25053040, PMID: 38474286 PMC10931698

[35] D'Alessio A Proietti G Sica G Scicchitano BM. Pathological and molecular features of glioblastoma and its Peritumoral tissue. Cancer. (2019) 11:469. doi: 10.3390/cancers11040469, PMID: 30987226 PMC6521241

[36] Beylerli O Talybov R Musaev E Trofimova T Shi H. Cerebrovascular disorders in patients with malignant tumors. Brain Hemorrhages. (2024) 5:284–92. doi: 10.1016/j.hest.2024.08.003

[37] Blystad I Warntjes JBM Smedby Ö Lundberg P Larsson EM Tisell A. Quantitative MRI for analysis of peritumoral edema in malignant gliomas. PLoS One. (2017) 12:e0177135. doi: 10.1371/journal.pone.0177135, PMID: 28542553 PMC5441583

[38] Bonosi L Marrone S Benigno UE Buscemi F Musso S Porzio M. Maximal safe resection in glioblastoma surgery: a systematic review of advanced intraoperative image-guided techniques. Brain Sci. (2023) 13:216. doi: 10.3390/brainsci13020216, PMID: 36831759 PMC9954589

[39] Zakharova NE Batalov AI Pogosbekian EL Chekhonin IV Goryaynov SA Bykanov AE. Perifocal zone of brain gliomas: application of diffusion kurtosis and perfusion MRI values for tumor invasion border determination. Cancer. (2023) 15:2760. doi: 10.3390/cancers15102760, PMID: 37345097 PMC10216555

[40] Grochans S Cybulska AM Simińska D Korbecki J Kojder K Chlubek D. Epidemiology of glioblastoma Multiforme–literature review. Cancer. (2022) 14:2412. doi: 10.3390/cancers14102412, PMID: 35626018 PMC9139611

[41] Mohammed S Dinesan M Ajayakumar T. Survival and quality of life analysis in glioblastoma multiforme with adjuvant chemoradiotherapy: a retrospective study. Reports Practical Oncol Radiotherapy. (2022) 27:1026–36. doi: 10.5603/RPOR.a2022.0113, PMID: 36632307 PMC9826661

[42] Sabouri M Dogonchi AF Shafiei M Tehrani DS. Survival rate of patient with glioblastoma: a population-based study. Egypt J Neurosurg. (2024) 39:42. doi: 10.1186/s41984-024-00294-5

[43] Fekete B Werlenius K Tisell M Pivodic A Smits A Jakola AS. What predicts survival in glioblastoma? A population-based study of changes in clinical management and outcome. Front Surg. (2023) 10:1249366. doi: 10.3389/fsurg.2023.1249366, PMID: 37711136 PMC10498299

[44] Jaoude DA Moore JA Moore MB Twumasi-Ankrah P Ablah E Moore DF Jr. Glioblastoma and increased survival with longer chemotherapy duration. Kansas J Med. (2019) 12:65–9. doi: 10.17161/kjm.v12i3.11795, PMID: 31489102 PMC6710024

[45] Mruk B. Renal safety of iodinated contrast media depending on their Osmolarity - current outlooks. Pol J Radiol. (2016) 81:157–65. doi: 10.12659/PJR.895406, PMID: 27141236 PMC4830331

[46] De Marco R Pesaresi A Bianconi A Zotta M Deandreis D Morana G. A systematic review of amino acid PET imaging in adult-type high-grade glioma surgery: a Neurosurgeon's perspective. Cancers (Basel). (2022) 15:90. doi: 10.3390/cancers15010090, PMID: 36612085 PMC9817716

[47] Yao Y Tan X Yin W Kou Y Wang X Jiang X. Performance of 18 F-FAPI PET/CT in assessing glioblastoma before radiotherapy: a pilot study. BMC Med Imaging. (2022) 22:226. doi: 10.1186/s12880-022-00952-w, PMID: 36566187 PMC9789562

[48] Ninatti G Pini C Bono BC Gelardi F Antunovic L Fernandes B. The prognostic power of [11C]methionine PET in IDH-wildtype diffuse gliomas with lower-grade histological features: venturing beyond WHO classification. J Neuro-Oncol. (2023) 164:473–81. doi: 10.1007/s11060-023-04438-9, PMID: 37695488

[49] Dadgar H Jokar N Nemati R Larvie M Assadi M. PET tracers in glioblastoma: toward neurotheranostics as an individualized medicine approach. Front Nucl Med. (2023) 3:1103262. doi: 10.3389/fnume.2023.1103262, PMID: 39355049 PMC11440984

[50] De Feo MS Granese GM Conte M. Immuno-PET for Glioma Imaging: An Update. Appl Sci. (2024) 14:1391. doi: 10.3390/app14041391, PMID: 40225413

[51] Robert JA Leclerc A Ducloie M Emery E Agostini D Vigne J. Contribution of [18F]FET PET in the Management of Gliomas, from diagnosis to follow-up: a review. Pharmaceuticals (Basel). (2024) 17:1228. doi: 10.3390/ph17091228, PMID: 39338390 PMC11435125

[52] Knudsen AM Halle B Cédile O Burton M Baun C Thisgaard H. Surgical resection of glioblastomas induces pleiotrophin-mediated self-renewal of glioblastoma stem cells in recurrent tumors. Neuro-Oncology. (2022) 24:1074–87. doi: 10.1093/neuonc/noab302, PMID: 34964899 PMC9248408

[53] Talybov R Beylerli O Mochalov V Prokopenko A Tatiana Ilyasova T Trofimova T. Multiparametric MR imaging features of primary CNS lymphomas. Front Surg. (2022) 9:887249. doi: 10.3389/fsurg.2022.887249, PMID: 35510125 PMC9058099

